# Supplementary material for: Extremely strong polarization of an active asteroid (3200) Phaethon
Source: Nat Commun. 2018 Jun 27;9:2486. doi: 10.1038/s41467-018-04727-2 (PMC6021421; doi:10.1038/s41467-018-04727-2)
Supplement: Supplementary file 1 — Supplementary Information [file 41467_2018_4727_MOESM1_ESM.pdf]

Supplementary Information

Extremely strong polarization of an active asteroid (3200) Phaethon

Ito *et al.*

Supplementary Table 1

The data used for plotting (1566) Icarus on Figure 1 in the main text.

α (deg)	P_r (%)	$P_{r,err}$ (%)
57.2	2.69	0.13
64.0	3.46	0.20
86.6	5.43	0.07
100.2	6.32	0.06
116.1	7.01	0.09
127.7	6.92	0.16
141.3	6.28	0.60

The values are based on Ref S1.

Supplementary Table 2

The data used for plotting (3200) Phaethon and (1566) Icarus on Figure 2 in the main text.

A	A_{err}	P_{max} (%)	$P_{max,err}$ (%)	object
0.091	0.006	49.8	-	Phaethon
0.075	-	49.8	-	Phaethon
0.060	-	49.8	-	Phaethon
0.215	0.018	7.32	0.25	Icarus

Phaethon's "P_{max}" is the largest observed value. The actual P_{max} is larger than this. Therefore, we did not assign any error values for this object. The data values for Icarus are based on Ref S1.

Supplementary Table 3

The data used for drawing Figure 3.

date (UT)	airmass	elevation (deg)	P_r (%)	$P_{r,err}$ (%)	unpolarized stars
2017-12-08	7.11	7.82	0.074	0.029	θ UMa
2016-10-01	1.75	34.78	0.062	0.031	HD 212311
2016-10-01	1.64	37.46	0.045	0.035	HD 212311
2016-10-01	1.52	41.22	0.057	0.031	HD 212311
2017-12-08	1.03	75.34	0.036	0.015	HD 212311

Supplementary Table 4

Phaethon's polarization degree P as a function of its relative rotation phase at each observation set. The tabulated values are used for drawing Supplementary Figure 1.

Panel (a)

date (UT)	time (UT)	P (%)	P_{err} (%)	rot phase	set #
2016-09-15	11:11:06	51.8	2.0	0.00000	1
2016-09-15	11:19:42	47.6	2.1	0.03977	2
2016-09-15	11:28:16	47.7	2.1	0.07939	3
2016-09-15	11:36:52	53.6	2.4	0.11916	4
2016-09-15.0.476		50.0	1.1	0.06659	night avr

Panel (b)

date (UT)	time (UT)	P (%)	P_{err} (%)	rot phase	set #
2016-09-17	15:31:57	52.2	2.5	0.52582	1
2016-09-17	15:45:09	42.0	2.1	0.58687	2
2016-09-17	15:57:44	51.5	2.0	0.64506	3
2016-09-17	16:10:19	48.0	1.8	0.70326	4
2016-09-17	18:16:50	43.0	3.7	0.28836	5
2016-09-17	18:25:24	50.6	2.0	0.32798	6
2016-09-17	18:34:00	48.4	1.3	0.36775	7
2016-09-17.0.708		48.1	0.7	0.93082	night avr

Panel (c)

date (UT)	time (UT)	P (%)	P_{err} (%)	rot phase	set #
2016-09-24	14:00:17	35.0	0.5	0.71844	1
2016-09-24	14:53:34	37.4	1.1	0.96485	2
2016-09-24	15:49:46	35.7	0.6	0.22476	3
2016-09-24.0.619		35.6	0.4	0.95467	night avr

Panel (d)

date (UT)	time (UT)	P (%)	P_{err} (%)	rot phase	set #
2016-10-04	15:43:13	20.8	0.2	0.78777	1
2016-10-04	15:49:50	20.1	0.5	0.81836	2
2016-10-04	15:56:25	18.9	1.5	0.84881	3
2016-10-04	16:19:45	20.2	0.3	0.95671	4
2016-10-04	16:32:58	18.3	1.3	0.01783	5
2016-10-04	16:44:28	20.5	0.2	0.07101	6
2016-10-04.0.675		20.4	0.2	0.92088	night avr

Panel (e)

date (UT)	time (UT)	P (%)	P_{err} (%)	rot phase	set #
2016-10-07	11:20:09	15.8	0.5	0.54880	1
2016-10-07	11:24:44	15.4	0.3	0.57000	2
2016-10-07	11:29:19	16.2	0.3	0.59119	3
2016-10-07	11:33:58	15.9	0.2	0.61269	4
2016-10-07	11:38:33	17.3	0.7	0.63389	5
2016-10-07	11:43:09	16.7	0.9	0.65516	6
2016-10-07	11:47:54	18.9	2.8	0.67713	7
2016-10-07	18:49:02	15.6	0.4	0.62462	8
2016-10-07	18:55:38	15.1	0.4	0.65514	9
2016-10-07	19:02:13	15.5	0.4	0.68559	10
2016-10-07	19:09:30	16.1	0.4	0.71927	11
2016-10-07	19:16:06	15.8	0.3	0.74979	12
2016-10-07.0.636		15.9	0.1	0.63874	night avr

Panel (f)

date (UT)	time (UT)	P (%)	P_{err} (%)	rot phase	set #
2016-11-07	10:27:41	3.1	0.8	0.73217	1
2016-11-07	10:36:17	4.4	1.3	0.77193	2
2016-11-07	10:44:53	9.6	4.5	0.81170	3
2016-11-07	12:39:49	5.4	1.0	0.34317	4
2016-11-07	12:54:49	2.2	1.5	0.41253	5
2016-11-07.0.485		3.5	0.6	0.05917	night avr

The nightly averaged values ("night avr") of P and P_{err} are equivalent to what are listed in Table 1. The relative rotation phases ("rot phase", between 0 and 1) are calculated from the rotation period of Phaethon (T_{rot} = 3.6039582 hours) published on Ref S2. The time when the first image set on the first night was obtained (2016-09-15 11:11:06 UT) is defined as the rotation phase = 0. The standard light-time correction is applied to the calculation of relative rotation phase. Note that in Panel (b) for 2016-09-17, P at rot phase = 0.58687 (set #2) has a lower value than others. We presume it is perhaps due to an intrusion of light from a nearby star when we applied aperture photometry to the asteroid images of this set.

Supplementary Figure1

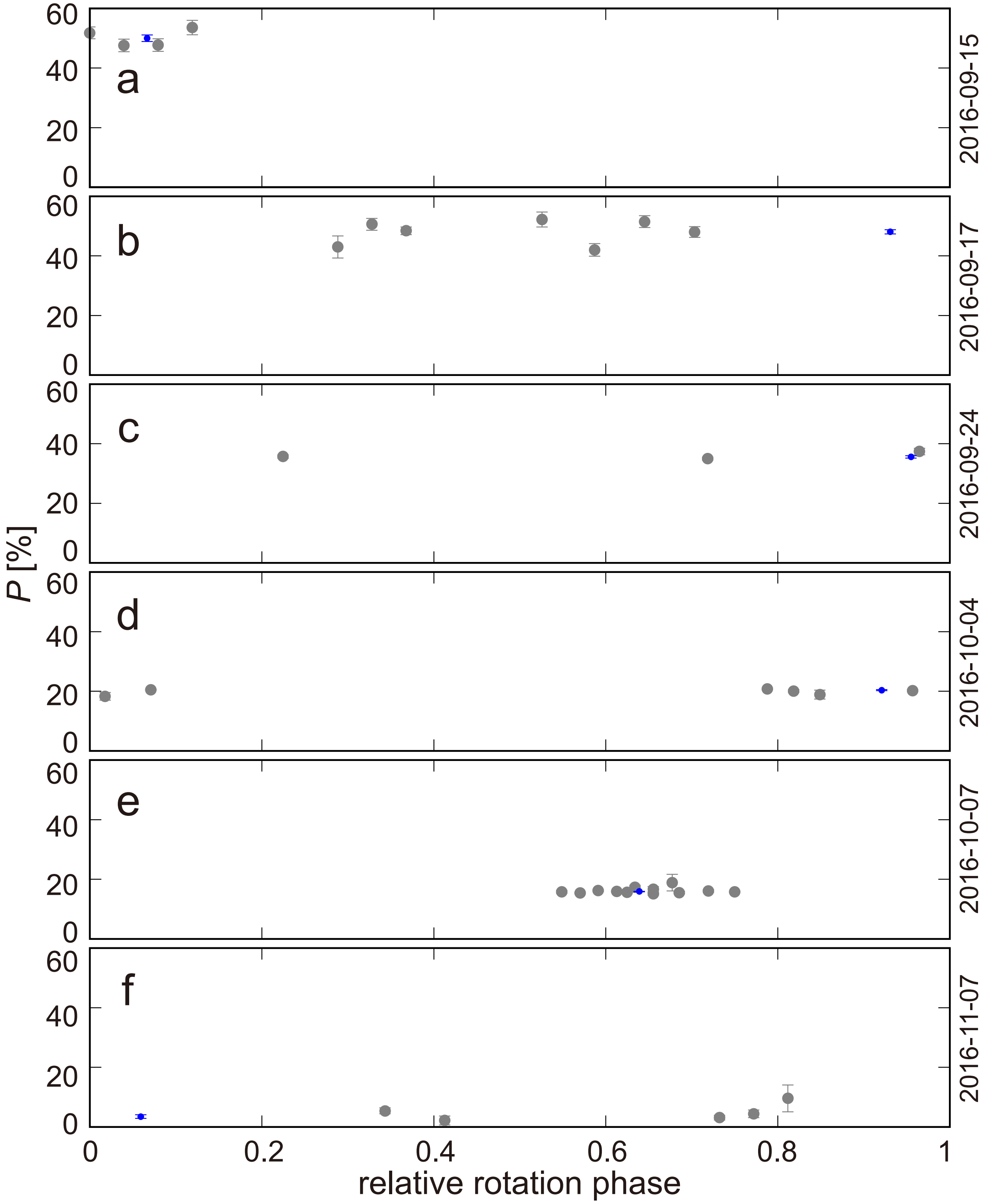

Supplementary Figure 1: See the next page for detailed caption.

Caption for Supplementary Figure 1. Linear polarization degree P of Phaethon as a function of its relative rotation phase at each observation night in 2016. a: September 15, b: September 17, c: September 24, d: October 4, e: October 7, and f: November 7. The gray-filled circles are the polarization degree measured at each of the image sets. As for the definition of the image "set", consult the Methods section in the main text. The blue-filled circles denote the nightly averages of the polarization degree for each night, equivalent to what are listed in Table 1. The relative rotation phases (between 0 and 1) are calculated from the rotation period of Phaethon ($T_{\rm rot} = 3.6039582$ hours) published on Ref S2. The time when the first image set was obtained on the first night (2016–09–15 11:11:06 UT) is defined as the rotation phase = 0. Error bars of P seen on the blue (nightly) symbols represent the sum of random errors and systematic errors that our polarimetric measurement contains. They are calculated through the inverse-variance weighting procedure (Ref S3) described in the Methods section, particularly in the Estimate of errors subsection. Error bars of P seen on the gray symbols represent the same kind of errors that each image set contains. The standard light–time correction is applied to the calculation of relative rotation phase. Supplementary Table 4 gives the actual data values used for this figure.

Supplementary References

S1. Ishiguro, M., Kuroda, D., Watanabe, M., Bach, Y. P., Kim, J., Lee, M., Sekiguchi, T., Naito, H., Ohtsuka, K., Hanayama, H., Hasegawa, S., Usui, F., Urakawa, S., Imai, M., Sato, M., and Kuramoto, K. Polarimetric study of near-Earth asteroid (1566) Icarus. *Astron. J.*, **154**, 180 (2017).

S2. Hanuš, J., Delbo', M., Vokrouhlický, D., Pravec, P., Emery, J. P., Alí-Lagoa, V., Bolin, B., Devogèle, M., Dyvig, R., Galád, A., Jedicke, R., Kornoš, L., Kušnirák, P., Licandro, J., Reddy, V., Rivet, J. -P., Világi, J., and Warner, B. D. Near-Earth asteroid (3200) Phaethon: Characterization of its orbit, spin state, and thermophysical parameters. *Astron. Astrophys.*, **592**, A34 (2016).

S3. Hartung, J., Knapp, G., and Sinha, B. K. *Statistical Meta-Analysis with Applications.* John Wiley & Sons, Hoboken, New Jersey (2008).
